# Non‐Cyclic Rozanolixizumab Administration in Complex Generalized Myasthenia Gravis

**DOI:** 10.1002/mus.70274

**Published:** 2026-05-20

**Authors:** Marc Abi Aoun, Diane Beauvais, Marlène Barnay, Marie‐Hélène Violleau, Stéphane Mathis, Fanny Duval, Guilhem Sole

**Affiliations:** ^1^ Neurology Department Robert Boulin Hospital Libourne France; ^2^ Neuromuscular Reference Center AOC, Neurology and Neuromuscular Diseases Department Pellegrin Hospital, Bordeaux University Hospitals Bordeaux France

**Keywords:** biomarkers, FcRn inhibition, myasthenia gravis, rozanolixizumab, steroid‐sparing therapy

## Abstract

**Introduction/Aims:**

Rozanolixizumab, an inhibitor of the neonatal Fc receptor (FcRn), is used to treat patients with generalized myasthenia gravis (gMG) refractory to standard therapies. However, the approved six‐week cyclic regimen may be associated with a “wearing‐off” effect and disease fluctuations. We evaluated the non‐cyclic administration of rozanolixizumab in patients with complex gMG.

**Methods:**

We retrospectively studied eight patients with anti–acetylcholine receptor antibody–positive complex gMG treated at Bordeaux University Hospital. “Complex” was defined by refractoriness, contraindications, intolerance to immunosuppressants or highly active MG. Patients transitioned to weekly or fortnightly rozanolixizumab after relapse during intermittent therapy. The primary outcome was ≥ 2‐point improvement in Myasthenia Gravis Activities of Daily Living (MG‐ADL) score at 3 months. Clinical data and serum immunoglobulin G (IgG) levels were collected at baseline, 3, 6, and 12 months.

**Results:**

The cohort (mean age 70.5 years; 75% female) had a mean baseline MG‐ADL score of 10.8, which improved rapidly to 4.1 at 3 months. Hospitalization days dropped from 15.8/month pre‐treatment to zero by 6–12 months. Corticosteroids were tapered from 30.3 to 13.3 mg/day at 1 year. Mean serum IgG levels declined by almost 50% and stabilized. Only one discontinuation occurred due to gastrointestinal symptoms; no serious infections or severe adverse events were observed.

**Discussion:**

Fortnightly rozanolixizumab yielded sustained clinical benefits, reduced hospitalizations, and enabled steroid tapering in complex gMG patients. This approach may prevent cyclical relapses seen with intermittent dosing. Larger studies are needed to confirm efficacy and long‐term safety.

**Abbreviations**
anti‐AChR: anti–acetylcholine receptor antibody
FcRn: neonatal Fc receptor
gMG: generalized myasthenia gravis
ICU: intensive care unit
IgG: immunoglobulin G
IVIg: intravenous immunoglobulins
M3/M6/M12: month 3/month 6/month 12
MG: myasthenia gravis
mg/d: milligrams per day
mg/dL: milligrams per deciliter
MG‐ADL: myasthenia gravis activities of daily living
MGFA: Myasthenia Gravis Foundation of America
PLEX: plasma exchange

## 1 Introduction

Some patients with generalized myasthenia gravis (gMG) remain difficult to control because of refractory [1] or highly active disease [2], leading to persistent fluctuations, recurrent hospitalizations, and prolonged corticosteroid exposure. These “complex” cases represent a major therapeutic challenge and highlight the need for alternative maintenance strategies.

Inhibition of the neonatal Fc receptor (FcRn) reduces pathogenic immunoglobulin G (IgG) levels and improves clinical outcomes in gMG [3]. Although rozanolixizumab is effective using an intermittent six‐week cyclic regimen [4, 5], symptom recurrence between cycles may occur in clinical practice, suggesting that sustained FcRn inhibition could be required in unstable disease, particularly in patients with severe or respiratory involvement.

We evaluated the clinical impact of weekly and fortnightly subcutaneous rozanolixizumab administration in patients with complex gMG who experienced relapse or treatment dependence during cyclic therapy.

## 2 Methods

### 2.1 Study Design and Participants

This retrospective case series included adult patients with anti–acetylcholine receptor antibody–positive (anti–AChR) gMG treated at Bordeaux University Hospital between November 2022 and December 2024 and recruited through review of medical records. Diagnosis required compatible clinical presentation associated with at least one confirmatory test (positive serology or decrement on repetitive nerve stimulation). “Complex” gMG included refractory disease according to published criteria [1] or highly active newly diagnosed disease requiring rapid treatment escalation [2]. None had prior FcRn or complement inhibitor therapy.

### 2.2 Rozanolixizumab Protocol

All patients initially received one or more standard 6‐week cycles of rozanolixizumab (7 mg/kg). Patients who experienced exacerbations within 4 weeks of the last injection were transitioned to a non‐cyclic regimen. Clinical exacerbation was defined as worsening of MG‐related symptoms requiring treatment escalation or MGFA class deterioration. In the initial patients, treatment initially consisted of weekly administration and was later adapted to a fortnightly schedule. Interval adjustments were guided by clinical stabilization and serum IgG levels, with administration postponed by 1 week when levels fell below 200 mg/dL. In later patients, early exacerbation led directly to initiation of the fortnightly regimen. An attempt to extend the dosing interval to every 3 weeks was performed to assess treatment dependence (Figure S1).

### 2.3 Data Collection and Study Endpoints

Clinical data were collected at baseline and months 3, 6, and 12. Baseline was defined at transition to the non‐cyclic regimen. The primary endpoint was ≥ 2‐point improvement in MG‐ADL at month 3 [4]. Secondary outcomes included hospitalization days, prednisone dose, serum IgG levels, Myasthenia Gravis Foundation of America (MGFA) class [6] and adverse events at Month 3 (M3), Month 6 (M6), and Month 12 (M12).

Because of the small sample size, analyses were descriptive and results are reported as means and ranges without formal statistical testing.

### 2.4 Protocol Approvals, Registrations, Patient Consent

All participants provided informed consent. Ethical approval was granted by the Health and Research Ethics Center of Bordeaux (approval #CER‐BDX‐2025‐278).

## 3 Results

### 3.1 Patient Demographics and Baseline Characteristics

Eight patients, all seropositive for anti–AChR antibodies and classified as MGFA class III–V were included (Table 1). Five patients had refractory disease and three had highly active newly diagnosed MG. Detailed characteristics are provided in the Table S1.

**TABLE 1:** Patient characteristics at baseline, M6 and M12.

	Baseline	M6	M12
Demographics			
Number of patients	8	8	8
Sex			
Female/male	6/2		
Age at inclusion, *y*			
Mean	70.5		
Range	49–94		
> 65, *n* (%)	6 (75%)		
BMI, kg/m^2^			
Mean	27.9		
Range	19.8–34.7		
MG characteristic			
MG duration, *y*			
Mean	6.3		
Range	0–23		
Serological status			
Anti‐AChR, *n* (%)	8 (100%)		
Thymus			
Thymectomy, *n* (%)	3 (37.5%)		
Thymoma, *n* (%)	3 (37.5%)		
Criteria for complex MG			
Refractoriness, *n* (%)	5 (62.5%)		
Highly‐active newly diagnosed MG, *n* (%)	3 (37.5%)		
MGFA class, *n* (%)			
Asymptomatic	0 (0%)	2 (25%)	3 (37.5%)
I	0 (0%)	3 (37.5%)	3 (37.5%)
IIa‐IIb	0 (0%)	3 (37.5%)	2 (25%)
IIIa‐IIIb	4 (50%)	0 (0%)	0 (0%)
IVa‐IVb	3 (37.5%)	0 (0%)	0 (0%)
V	1 (12.5%)	0 (0%)	0 (0%)
MG‐ADL score			
Mean	10.8	3.6	3.1
Range	6–16	1–8	1–7
Monthly hospitalization days			
mean	15.75	0	0
Medications			
Prednisone			
*n* (%)	7 (87.5%)	6 (75%)	6 (75%)
Mean dose, mg/d	30.3	16.8	13.3
Range, mg/d	0–60	0–20	0–17.5
Rescue therapies in the past 2 months			
IVIg *n* (%)	4 (50%)	0 (0%)	0 (0%)
PLEX *n* (%)	3 (37.5%)	0 (0%)	0 (0%)
Serum IgG level, mg/dL			
Mean	690	340	390
Range	130–1120	170–430	180–550

Abbreviations: Anti‐AChR, anti‐acetylcholine receptor antibody; BMI, body mass index; IVIg, intravenous immunoglobulins; kg/m^2^, kilograms per square meter; M12, month 12; M6, month 6; mg/d, milligrams per day; mg/dL, milligrams per deciliter; MG, myasthenia gravis; MG‐ADL, myasthenia gravis activities of daily living; MGFA, Myasthenia Gravis Foundation of America; *n*, number; PLEX, plasma exchange; Serum IgG, serum immunoglobulin G level; y, year.

### 3.2 Clinical Outcomes

**FIGURE 1:**
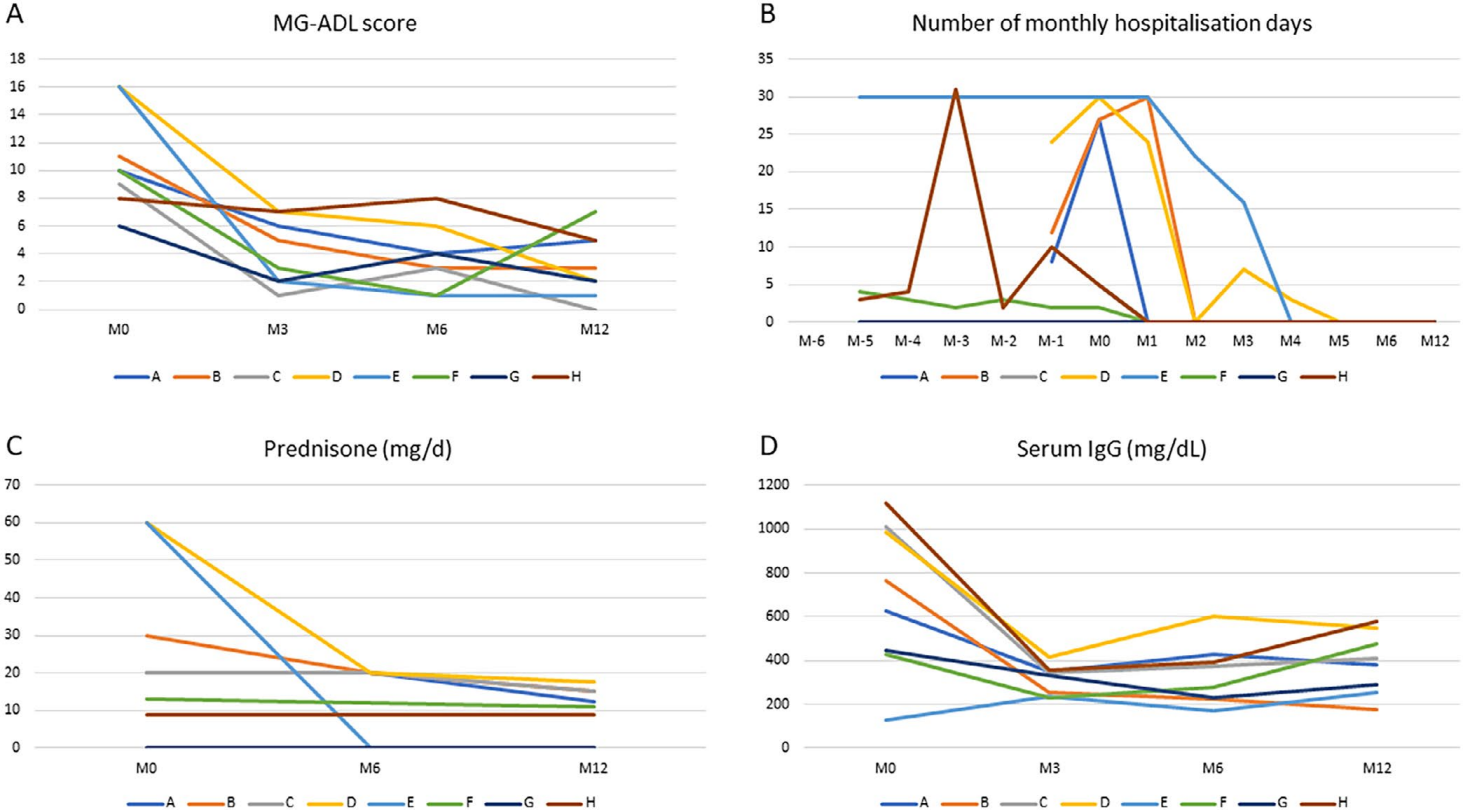
(A–D) Evolution on rozanolixizumab non‐cyclic administration. (A) MG‐ADL (Myasthenia Gravis Activities of Daily Living) score from baseline to M12. (B) Number of monthly hospitalization days. Lines starts after M‐5 for newly diagnosed patients. (C) Daily prednisone dose in milligrams per day from baseline to M12. (D) Serum Immunoglobulin G level in grams per liter from baseline to M12. Each line represents an individual patient (A–H). Overlapping lines may not be individually distinguishable, giving the impression of a single line.

MG‐ADL scores improved from a mean baseline of 10.8–4.1 at M3 and stabilized at 3.1 at M6 and M12 (Figure 1A). All patients achieved a ≥ 2‐point improvement except one 94‐year‐old patient, whose score remained stable. In her case, azathioprine had been discontinued because of recurrent urinary tract infections, resulting in disease instability requiring IVIg and hospitalizations for heart failure; no further treatment‐related complications occurred after initiation of rozanolixizumab. MGFA class improved from ≥ III to ≤ II in all patients.

### 3.3 Rozanolixizumab Administration, Hospitalization Impact, and Steroid Tapering

Monthly hospitalization days markedly decreased, reaching zero or near zero in all patients, including patients with high baseline hospitalization rates (≥ 25–30 days/month; Figure 1B). Pre‐treatment hospitalization patterns reflected two distinct clinical profiles. Four patients had severe disease requiring prolonged hospitalization, including one prolonged intensive care unit (ICU) admission for refractory myasthenic crisis and two patients requiring enteral feeding despite intravenous immunoglobulin (IVIg) and plasma exchange (PLEX). These three patients improved after cyclic regimen but experienced relapse approximately 3 weeks after treatment, leading to intensification to weekly and subsequently fortnightly dosing because of recurrent low serum IgG levels. One additional patient with highly active, newly diagnosed disease was initially admitted to the ICU, discharged after the first cycle, and required a second cycle at day 18 because of bulbar worsening, prompting a switch to fortnightly treatment. The four remaining patients had less severe disease (MGFA IIIA–IIIB) with limited or no prior hospitalization and transitioned to fortnightly dosing because of rozanolixizumab dependence. An attempt to extend the dosing interval to every 3 weeks was performed in all patients after initial stabilization but resulted in clinical worsening, confirming treatment dependence and supporting continuation of the fortnightly regimen. Corticosteroids were discontinued in one patient and reduced in all remaining treated patients (Figure 1C).

### 3.4 Serum IgG Levels and Safety

Consistent with FcRn inhibition, serum IgG levels decreased rapidly and remained stable (Figure 1D). Among patients initially treated with weekly injections, approximately 30% of administrations were postponed because of serum IgG levels < 200 mg/dL, whereas only three administrations were deferred after initiation of the fortnightly regimen. Adverse events included upper respiratory tract infections (*n* = 7), COVID‐19, gastroenteritis, urinary tract infection, genital fungal infection, and chronic obstructive pulmonary disease exacerbation (all *n* = 1). No infection was severe or led to MG exacerbation, hospitalization, or treatment discontinuation. Myalgia (*n* = 1), headache (*n* = 2), and diarrhea (*n* = 3) occurred during initial injections and resolved, except in one patient with ulcerative colitis who discontinued treatment after 1 year because of persistent diarrhea. One myocardial infarction was reported and deemed unrelated to treatment.

## 4 Discussion

In this case series of patients with complex anti–AChR–positive gMG, fortnightly subcutaneous rozanolixizumab was associated with rapid and sustained clinical improvement, reduced hospitalization burden, and corticosteroid tapering.

Our findings align with the MycarinG trial, in which cyclical rozanolixizumab provided rapid symptom relief; however, discontinuation after each cycle may lead to symptom rebound, thereby requiring initiation of a subsequent cycle [5]. In contrast, a non‐cyclic regimen is designed to mitigate clinically relevant “wearing‐off” effect through sustained FcRn inhibition, an approach that may be particularly relevant in highly active disease or in patients with severe manifestations, including respiratory involvement. The sustained control observed here supports the hypothesis that selected patients may benefit from non‐cyclic FcRn inhibition to prevent clinically relevant “wearing‐off” effect, consistent with the MG0004 extension study using weekly dosing [7].

Our cohort reflects a “complex gMG” population extending beyond the conventional definition of refractory MG [1, 8]. Although refractory MG implies insufficient response to adequately dosed standard immunotherapies, intolerance, or contraindications, often with persistent need for rescue therapy, our definition also included patients with highly active disease at onset requiring early treatment escalation before adequate exposure to standard agents [2]. This distinction suggests that therapeutic refractoriness and initial disease severity represent related but distinct aspects of disease complexity, both associated with instability and unmet clinical need. This broader framework is clinically relevant, as similar complex profiles are frequently encountered in referral centers, where treatment escalation is frequently limited by multimorbidity, polypharmacy, and safety concerns rather than lack of immunologic responsiveness. A notable feature of our cohort was advanced age, exceeding that of pivotal anti‐FcRn trials [4, 9, 10]. Although MG is not necessarily more severe in older patients [11], a subset is characterized by greater comorbidity burden, polypharmacy, and competing risks influencing management and prognosis [12, 13]. In our series, therapeutic options were often constrained by comorbidities, supporting the clinical relevance of alternative maintenance strategies.

Rozanolixizumab was well tolerated, with serum IgG levels decreasing by approximately 50%. Treatment deferral was protocol‐driven according to IgG levels. Frequent postponements during weekly dosing suggest pharmacodynamic overexposure, whereas transition to a fortnightly regimen was associated with improved IgG level stability and sustained clinical control, supporting a more appropiate maintenance schedule. The higher postponement rate during weekly dosing may reflect baseline immune vulnerability related to prior immunosuppressive exposure, including rituximab. Despite these pharmacodynamic effects, no severe infections occurred, consistent with pooled safety data from FcRn inhibitor trials reporting serious infections in fewer than 5% of cases [10, 14, 15].

This study has several limitations, including the small sample size, single‐center design, restricted patient spectrum (older, anti–AChR–positive), and observational design without a control group, which limit generalizability and causal inference. The cohort also represents an enriched responder population, as non‐responders to the initial cycle were not redosed, potentially overestimating treatment benefit. Larger multicenter studies including broader patient populations are needed to confirm these findings.

In conclusion, fortnightly rozanolixizumab may represent a maintenance strategy for selected patients with complex gMG, particularly in those experiencing clinically relevant “wearing‐off” effect with standard cyclic regimens and failure to achieve sustained disease control.

## Author Contributions

**Marc Abi Aoun:** investigation, writing – review and editing, writing – original draft. **Guilhem Sole:** conceptualization, investigation, writing – review and editing, writing – original draft, methodology, validation, formal analysis. **Fanny Duval:** investigation, writing – review and editing. **Stéphane Mathis:** writing – review and editing. **Marlène Barnay:** investigation, writing – review and editing. **Diane Beauvais:** investigation, writing – review and editing. **Marie‐Hélène Violleau:** conceptualization, methodology, data curation, formal analysis.

## Funding

The authors have nothing to report.

## Ethics Statement

Ethical approval was obtained from the Health and Research Ethics Center of Bordeaux (approval #CER‐BDX‐2025‐278). We confirm that we have read the Journal's position on issues involved in ethical publication and affirm that this report is consistent with those guidelines.

## Consent

According to French regulations, all participants provided informed consent for data collection and publication.

## Conflicts of Interest

Dr. Guilhem Solé has received honoraria from UCB for board participation and educational activities. The other authors declare no conflicts of interest.

## Supporting information


**Figure S1:** mus70274‐sup‐0001‐Figure.jpg.

**Table S1:** Summary of Patient Characteristics and Indications for Continuous Rozanolixizumab Administration.

## Data Availability

The authors confirm that anonymized data supporting the findings of this study will be made available upon reasonable request to qualified investigators.
