# Supplementary figures and images for: Non‐Cyclic Rozanolixizumab Administration in Complex Generalized Myasthenia Gravis
Source: Muscle Nerve. 2026 May 20;74(2):422–6. doi: 10.1002/mus.70274 (PMC13332567; doi:10.1002/mus.70274)

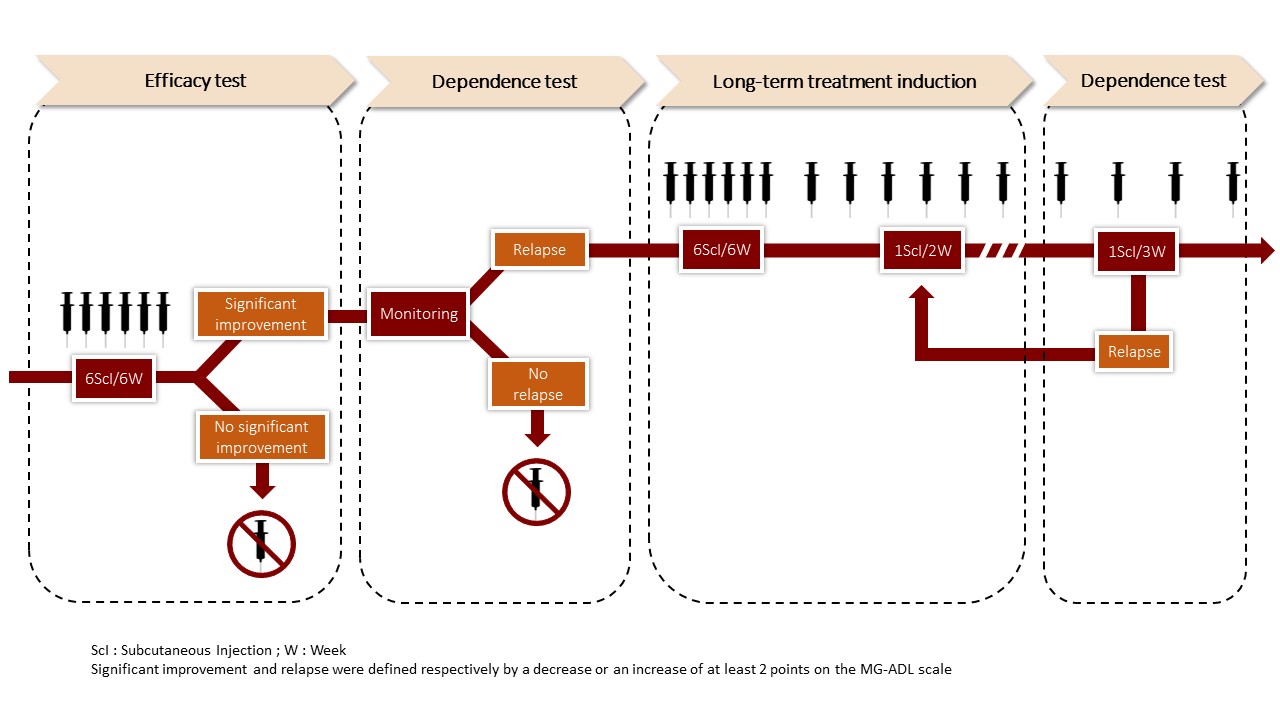

Supplement: Supplementary file 1 — Figure S1: mus70274‐sup‐0001‐Figure.jpg. [file MUS-74-422-s002.jpg]
