# Supplementary material for: Non‐Cyclic Rozanolixizumab Administration in Complex Generalized Myasthenia Gravis
Source: Muscle Nerve. 2026 May 20;74(2):422–6. doi: 10.1002/mus.70274 (PMC13332567; doi:10.1002/mus.70274)
Supplement: Supplementary file 2 — Table S1: Summary of Patient Characteristics and Indications for Continuous Rozanolixizumab Administration. [file MUS-74-422-s001.docx]

**Supplementary Table. Summary of Patient Characteristics and Indications for Continuous Rozanolixizumab Administration**

| Patient | Age (years) | Sex | Disease duration | Thymic Abnormalities (Treatment) | Major Comorbidities | Long-Term Therapies | Rescue Therapies | Indication for Continuous Treatment |
| --- | --- | --- | --- | --- | --- | --- | --- | --- |
| A | 70 | M | 23 y | Thymoma (surgery) | COPD; diabetes mellitus; recurrent respiratory and urinary infections; recurrent mycotic infections | CS (20 y); MMF (14 mo, discontinued—lack of efficacy); AZA (10 y, discontinued—recurrent infections) | IVIg (effective, repeated use); PLEX (not used) | Discontinuation of CS/AZA due to recurrent mycotic infections |
| B | 75 | F | 10 mo | None | None | CS (9 mo, ongoing); AZA (early discontinuation—hepatotoxicity); RTX (9 mo, ongoing) | IVIg (partial response); PLEX (dependence) | Highly active refractory MG and bulbar involvement requiring nasogastric feeding, later gastrostomy |
| C | 62 | M | 1 y | None | Lung neoplasm; recurrent infections | CS (13 mo, ongoing); Other immunosuppressants contraindicated (lung neoplasm) | IVIg (dependence)  PLEX (not used) | Highly active MG with inability to taper CS and IVIg dependence |
| D | 49 | F | 3 mo | None | Long-standing type 1 diabetes mellitus; obesity | CS (2 mo, ongoing); RTX (1 single infusion, discontinued—severe allergic reaction) | IVIg (ineffective); PLEX (ineffective) | Highly active early-onset MG with no alternative therapy, requiring ICU management |
| E | 72 | F | 2 y | Thymoma type B (surgery + RT) | Graves’ disease; systemic sclerosis (no visceral involvement) | CS (2 y, ongoing); AZA (1 y, discontinued—lack of efficacy); RTX (1 y, ongoing) | IVIg (ineffective); PLEX (ineffective) | Relapses despite maximal therapy requiring ICU admission and invasive ventilation |
| F | 72 | F | 7 y | Thymoma type AB, stage III (surgery + RT) | Lymphopenia; atypical mycobacterial infection; myocardial infarction | CS (10 y, ongoing); RTX (2 y, discontinued—lack of efficacy and infection) | IVIg (dependence); PLEX (not used) | Inability to taper CS and IVIg dependence |
| G | 70 | F | 15 y | None | None | CS (6 mo, discontinued—neuropsychiatric adverse effects); AZA (3 y, discontinued—lymphopenia); MMF (6 y) | IVIg (dependence)  PLEX (not used) | Inclusion in MyCaring study; rozanolixizumab dependence with short dosing interval (15 days) |
| H | 94 | F | 3 y | None | Heart failure; renal failure; recurrent UTI; poor venous access | CS (3 y, ongoing at low dose due to heart failure); AZA (14 mo- discontinued—recurrent UTI infections) | IVIg (good response but recurrent heart failure); IVIg dependence  PLEX (not feasible) | IVIg dependence with recurrent acute heart failure episodes |

**Abbreviations:** AZA = azathioprine; COPD = chronic obstructive pulmonary disease; CS = corticosteroids; ICU = intensive care unit; IVIg = intravenous immunoglobulin; MMF = mycophenolate mofetil; PLEX = plasma exchange; RT = radiotherapy; RTX = rituximab; UTI = urinary tract infection; y = years; mo = months.
